# Supplementary material for: Three-year functional, physical, and mental health outcomes after critical COVID-19: A prospective multicentre cohort study
Source: PLoS One. 2026 Feb 18;21(2):e0341319. doi: 10.1371/journal.pone.0341319 (PMC12915914; doi:10.1371/journal.pone.0341319)
Supplement: S1 Table — Descriptive comparison of demographic and clinical characteristics between retained and lost participants. (DOCX) [file pone.0341319.s001.docx]

**Supplementary Table 1. Comparison of participants retained in the 3-year follow-up and those lost between 1 and 3 years.**

| **Variable** | **Retained at 3 years (n = 191)** | **Lost to follow-up (n = 19)** |
| --- | --- | --- |
| Age | 62 [53–69] | 62 [56–69] |
| Male (%) | 73 | 61 |
| Native Swedish speaker (%) | 63 | 47 |
| SAPS 3 | 57 [48–66] | 60 [46–67] |
| BMI | 31 [28–35] | 28 [26–38] |
| CFS | 3 [2–3] | 3 [3–3] |
| CCI | 2 [1–3] | 2 [1–3] |
| GOSE at 1 year | 7 [6–8] | 6 [5–7] |

*Values are median [IQR] unless otherwise stated. Because the number of dropouts was small (n < 20), comparisons are presented descriptively. Individuals lost to follow-up were more often non-native Swedish speakers and less frequently male, and had slightly higher SAPS 3 scores, lower BMI, and lower functional outcome at 1 year. No meaningful differences were observed in comorbidity burden or frailty. Abbreviations: BMI = Body Mass Index; CCI = Charlson Comorbidity Index; CFS = Clinical Frailty Scale; SAPS 3 = Simplified Acute Physiology Score 3; GOSE = Glasgow Outcome Scale Extended.*
