# Supplementary material for: Three-year functional, physical, and mental health outcomes after critical COVID-19: A prospective multicentre cohort study
Source: PLoS One. 2026 Feb 18;21(2):e0341319. doi: 10.1371/journal.pone.0341319 (PMC12915914; doi:10.1371/journal.pone.0341319)
Supplement: S2 Table — GOSE was analysed using the Kruskal–Wallis test, and PCS and MCS using one-way analysis of variance. (DOCX) [file pone.0341319.s002.docx]

**Supplementary Table 2. Functional outcome and Health-Related Quality of Life at 3 years, stratified by age group**

| **Outcome measure** | **<50 years (n = 37)** | **50–65 years (n = 81)** | **≥65 years (n = 72)** | **p-value** |
| --- | --- | --- | --- | --- |
| GOSE, median (IQR) | 6 [6–7] | 6 [6–7] | 7 [6–7] | **0.036** |
| PCS, mean (95% CI) | 46.8 (42.9–50.6) | 44.6 (42.4–46.7) | 44.4 (42.7–46.2) | 0.459 |
| MCS, mean (95% CI) | 45.3 (41.8–48.8) | 45.0 (41.9–48.1) | 45.9 (43.1–48.8) | 0.895 |

*GOSE, Glasgow Outcome Scale Extended; PCS, Physical Component Summary of the SF-36v2®; MCS, Mental Component Summary of the SF-36v2®; IQR, interquartile range; CI, confidence interval. Age groups were defined as <50, 50–65, and ≥65 years. GOSE was analysed using the Kruskal–Wallis test due to its ordinal scale. PCS and MCS were analysed using one-way ANOVA. Statistically significant p-values (two-sided) are shown in bold (p < 0.05).*

|  |  |  |
| --- | --- | --- |
|  |  |  |
|  |  |  |
|  |  |  |
|  |  |  |
|  |  |  |
|  |  |  |
|  |  |  |
|  |  |  |
