# Supplementary material for: Three-year functional, physical, and mental health outcomes after critical COVID-19: A prospective multicentre cohort study
Source: PLoS One. 2026 Feb 18;21(2):e0341319. doi: 10.1371/journal.pone.0341319 (PMC12915914; doi:10.1371/journal.pone.0341319)
Supplement: S3 Table — Descriptive results for all subdomains of SF-36v2, HADS, PCL-5, MFIS, and SGRQ at 1- and 3-year follow-up. (DOCX) [file pone.0341319.s003.docx]

Supplementary Table 3. Subdomains of patient-reported outcome measures at 1 and 3 years.

| Outcome measure | 1-year (n=191) | 3-year (n=191) | Mean difference  and (MID) | p-value |
| --- | --- | --- | --- | --- |
| Subdomains of SF-36v2^®^ (HRQoL) (n=146) | | | | |
| Physical Functioning | 43.8 (42.3-45.4) | 43.7 (42.2-45.2) | -0.12 (3) | 0.910 |
| Role-Physical | 43.6 (42.0-45.3) | 41.8 (40.3-43.2) | -1.85 (3) | 0.014** |
| Bodily Pain | 46.7 (44.9-48.5) | 45.3 (43.8-46.9) | -1.41 (3) | 0.110 |
| General Health | 46.0 (44.2-47.7) | 45.9 (44.2-47.6) | -0.08 (2) | 0.700 |
| Vitality | 47.9 (46.1-49.7) | 54.0 (52.2-55.8) | 6.07 (2)* | <0.001** |
| Social Functioning | 47.1 (45.4-48.8) | 41.1 (39.4-42.7) | -6.01 (3)* | <0.001** |
| Role-Emotional | 44.8 (42.8-46.7) | 42.9 (41.0-44.8) | -1.91 (4) | 0.014** |
| Mental Health | 49.7 (47.9-51.4) | 43.9 (42.2-45.6) | -5.77 (3)* | <0.001** |
| Subdomains of MFIS (n=144) | | | | |
| Physical (0-36), mean (95% CI) | 15.8 (14.4-17.2) | 17.3 (15.7-18.9) | NA | 0.020** |
| Cognitive (0-40), mean (95% CI) | 13.0 (11.5-14.4) | 14.4 (12.8-15.9) | NA | 0.015** |
| Psychosocial (0-8), mean (95% CI) | 2.8 (2.4-3.1) | 3.0 (2.6-3.4) | NA | 0.030** |
| Subdomains of SGRQ (Respiratory function) (n=145) | | | | |
| Symptoms (0-100), mean (95% CI) | 28.0 (24.4-31.7) | 31.0 (27.0-35.0) | 3.0 (4.0) | 0.021** |
| Activity (0-100), mean (95% CI) | 44.2 (39.6-48.8) | 44.5 (40.0-48.9) | -0.3 (4.0) | 0.200 |
| Impacts (0-100), mean (95% CI) | 20.0 (16.7-23.2) | 20.9 (17.3-24.5) | 0.9 (4.0) | 0.520 |
| Total (0-100), mean (95% CI) | 29.3 (25.8-32.9) | 29.7 (26.1-33.4) | 0.4 (4.0) | 0.350 |

Values from SF-36v2^®^ are presented as T-scores of the mean (95% CI). All other values are presented as mean (95% CI). MID values in parentheses represent established minimally important differences for each measure. MIDs for subdomains of MFIS are not well-established. MID = Minimal Important Difference; CI = Confidence Interval; HRQoL = Health-Related Quality of Life; SF-36v2^®^ = 36-Item Short Form Survey; MFIS = Modified Fatigue Impact Scale; SGRQ = St. George's Respiratory Questionnaire.

*above MID

**statistically significant p=<0.05
