# Supplementary material for: Three-year functional, physical, and mental health outcomes after critical COVID-19: A prospective multicentre cohort study
Source: PLoS One. 2026 Feb 18;21(2):e0341319. doi: 10.1371/journal.pone.0341319 (PMC12915914; doi:10.1371/journal.pone.0341319)
Supplement: S4 Table — Odds ratios, 95% confidence intervals, and p-values for each patient-reported outcome measure. (DOCX) [file pone.0341319.s004.docx]

Supplementary Table 4. Univariable logistic regression analysis of patient-reported outcomes associated with incomplete recovery (GOSE ≤ 6) at 3 years

|  | | | |
| --- | --- | --- | --- |
|  | OR | p-value |  |
| Life Satisfaction | 0.66 [0.55-0.79] | <0.001* |  |
| PCS | 0.90 [0.85-0.95] | <0.001* |  |
| MCS | 0.92 [0.88-0.95] | <0.001* |  |
| Physical Functioning | 0.93 [0.89-0.97] | <0.001* |  |
| Role-Physical | 0.93 [0.89-0.97] | <0.001* |  |
| Bodily Pain | 0.92 [0.88-0.96] | <0.001* |  |
| General Health | 0.92 [0.88-0.95] | <0.001* |  |
| Vitality | 0.89 [0.86-0.93] | <0.001* |  |
| Social Functioning | 0.90 [0.87-0.94] | <0.001* |  |
| Role-Emotional | 0.95 [0.92-0.98] | <0.001* |  |
| Mental Health | 0.91 [0.87-0.94] | <0.001* |  |
| HADS A | 1.22 [1.12-1.33] | <0.001* |  |
| HADS D | 1.25 [1.13-1.38] | <0.001* |  |
| MFIS | 1.06 [1.04-1.09] | <0.001* |  |
| PCL-5 | 1.05 [1.03-1.08] | <0.001* |  |
| SGRQ | 1.04 [1.02-1.06] | <0.001* |  |

OR = Odds Ratio with 95% confidence intervals in brackets. GOSE = Glasgow Outcome Scale Extended; PCS = Physical Component Summary; MCS = Mental Component Summary from SF-36v2; HADS A = Hospital Anxiety and Depression Scale-Anxiety; HADS D = Hospital Anxiety and Depression Scale-Depression; MFIS = Modified Fatigue Impact Scale; PCL-5 = PTSD Checklist for DSM-5; SGRQ = St. George's Respiratory Questionnaire.

*statistically significant p=<0.05
