# Supplementary material for: Three-year functional, physical, and mental health outcomes after critical COVID-19: A prospective multicentre cohort study
Source: PLoS One. 2026 Feb 18;21(2):e0341319. doi: 10.1371/journal.pone.0341319 (PMC12915914; doi:10.1371/journal.pone.0341319)
Supplement: S5 Table — Correlation matrix showing relationships between GOSE, PCS, MCS, SGRQ, fatigue scores, and psychological symptom measures. (DOCX) [file pone.0341319.s005.docx]

Supporting Table 5. Spearman correlation coefficients among clinical outcome measures and Patient-Reported Outcome Measures at 3 years

|  | GOSE | HADS A | HADS D | PCL-5 | MFIS | SGRQ | PCS | MCS |
| --- | --- | --- | --- | --- | --- | --- | --- | --- |
| HADS A | -0.38** |  |  |  |  |  |  |  |
| HADS D | -0.43** | 0.75** |  |  |  |  |  |  |
| PCL-5 | -0.40** | 0.73** | 0.74** |  |  |  |  |  |
| MFIS | -0.53** | 0.56** | 0.73** | 0.71** |  |  |  |  |
| SGRQ | -0.45** | 0.51** | 0.56** | 0.56** | 0.64** |  |  |  |
| PCS | 0.48** | -0.22** | -0.35** | -0.34** | -0.56** | -0.65** |  |  |
| MCS | 0.45** | -0.66** | -0.71** | -0.65** | -0.70** | -0.51** | 0.41** |  |
| Life satisfaction | 0.42** | -0.46** | -0.54** | -.45** | -0.52** | -0.43** | 0.43** | 0.63** |

GOSE = Glasgow Outcome Scale Extended; HADS A = Hospital Anxiety and Depression Scale - Anxiety subscale; HADS D = Hospital Anxiety and Depression Scale - Depression subscale; PCL-5 = PTSD Checklist for DSM-5; MFIS = Modified Fatigue Impact Scale; SGRQ = St. George's Respiratory Questionnaire; PCS = Physical Component Summary (SF-36);, MCS = Mental Component Summary (SF-36). Correlation coefficients ≥ 0.50 indicate strong correlations, 0.30-0.49 indicate moderate correlations, and 0.10-0.29 indicate weak correlations. ** indicates p < 0.001
