# Supplementary material for: Three-year functional, physical, and mental health outcomes after critical COVID-19: A prospective multicentre cohort study
Source: PLoS One. 2026 Feb 18;21(2):e0341319. doi: 10.1371/journal.pone.0341319 (PMC12915914; doi:10.1371/journal.pone.0341319)
Supplement: Attachment — Submitted filename: PONE review.pdf [file pone.0341319.s006.pdf]

I thank the Editor for the opportunity to review the manuscript “Three-year functional, physical, and mental health outcomes after critical COVID-19: A prospective multicentre cohort study” by Dr Didriksson and colleagues. In the manuscript the authors report results from a follow-up telephone assessment of patients that have survived critical COVID-19 three years previously, and compare the results with previous assessments of the same cohort. The results are interesting, and the authors should be commended for the focus on patient-important outcomes and good retention of the cohort. The main result was that at three years, the patients´ functional outcome had impaired compared to the one-year follow-up. This is unsettling, as especially younger age, but also frailty, appears as a risk factor for deterioration. The study has several merits, such as use of validated instruments by trained assessors, and a small number of dropouts. The manuscript is well written, easy to follow, and the figures and tables are clear. I have, however, some questions regarding the study and interpretation of the results that need clarification.

1. The one-year mortality in the original cohort seems very high (48%), when compared with the 30-d mortality of 27% in Sweden, reported in a study by Chew et al [1]. Can the authors discuss this and how a potentially higher than average mortality and probably disease severity, affect the generalizability of the results?
2. Although the retention was good, almost 10% of the patients dropped out between 1- and 3-year follow-ups. Did the authors assess who these drop-outs were?
3. There was a considerable number of non-native Swedish speaking patients in the cohort, how does this affect the results? Do the population norms fit well for immigrants? Are there cultural or language-related aspects in interpreting the results of the questionnaires? Do the population norms fit well in analysing results in immigrant populations?
4. How would the different assessment methods, face-to-face and telephone assessment affect the results? The authors have listed this as a limitation, but since the result of impairment in the results is not unsurprising, it would be valuable to discuss the differences in more detail.
5. One important issue is that there are no controls in this study. Pandemic affected the whole society in most countries in many different ways, which may compromise population norms as a standard, because also individuals without critical covid or no covid at all may have experienced challenges in mental health. Mirroring the results of this cohort to contemporary controls might have given different, less dramatic impairment. Please discuss. I am aware that a control group with no COVID-19 at all would be almost impossible to obtain, but the challenges of a design with no controls should be acknowledged.
6. In intensive care literature on long-term follow-up there is an inherent challenge that the connection to the initial event (here COVID-19) becomes weaker as time passes, and other life events occur and confound the results. In this study, too, it is possible that the patients experience life events that are not at all related to the COVID-19. Also here, the control group would be helpful. It would be important to discuss also this aspect, that it is not clear how much these results are due to history of covid, or are they possibly a result of other challenges, such as aging, other diagnoses, etc.
7. Did the authors record any psychiatric diagnoses at baseline? This would be important information for interpreting the results.
8. Some of the patients were still receiving rehabilitation at three years. How were patients supported if they were found having psychiatric symptoms at one-year follow-up? Did they receive support, and could this have affected the results?

Ref.

1. Chew MS, Kattainen S, Haase N, Buanes EA, Kristinsdottir LB, Hofsø K, Laake JH, Kvåle R, Hästbacka J, Reinikainen M, Bendel S, Varpula T, Walther S, Perner A, Flaatten HK, Sigurdsson MI. A descriptive study of the surge response and outcomes of ICU patients with COVID-19 during

first wave in Nordic countries. Acta Anaesthesiol Scand. 2022 Jan;66(1):56-64. doi: 10.1111/aas.13983. Epub 2021 Oct 3. PMID: 34570897; PMCID: PMC8652908.
