# Supplementary material for: Three-year functional, physical, and mental health outcomes after critical COVID-19: A prospective multicentre cohort study
Source: PLoS One. 2026 Feb 18;21(2):e0341319. doi: 10.1371/journal.pone.0341319 (PMC12915914; doi:10.1371/journal.pone.0341319)
Supplement: Attachment — Submitted filename: Response_to_Reviewers.docx [file pone.0341319.s008.docx]

# RESPONSE TO REVIEWERS

**PONE-D-25-38284**
**Three-year functional, physical, and mental health outcomes after critical COVID-19: A prospective multicentre cohort study**

*We thank the Editor and the Reviewers for their constructive feedback. We have carefully addressed all comments and revised the manuscript accordingly. Below, we provide point-by-point responses to each comment, with references to the corresponding changes in the revised manuscript.*

## JOURNAL REQUIREMENTS

### 1. File naming and style requirements

***Response:*** *We have updated all file names to follow PLOS ONE conventions (Manuscript.docx, S1–S4 Tables, and Fig 1–7, submitted in approved formats).*

### 2. Grant information mismatch

***Response:*** *We are thankful that this discrepancy has been noted. We have now reviewed and aligned all funding information across the submission system and the manuscript. The Funding Information section has been updated with the correct grant numbers. Also, the Funding/ Financial Disclosure statement in the manuscript has been revised to match these entries.*

### 3. Data availability contact point

***Response:*** *The Data Availability statement has been revised. Region Skåne, as the institutional data owner, is now listed as the non-author contact point in accordance with PLOS ONE data policy.*

### 4. Ethics statement placement

***Response:*** *The ethics statement has now been removed from the Declarations section and appears only in the Materials and Methods section, in accordance with PLOS ONE requirements*.

### 5. Supplementary tables

***Response:*** *All supplementary tables have now been removed from the main manuscript body and provided as separate Supporting Information files. Each file has been named according to PLOS ONE conventions (S1–S4 Table), and the corresponding legends have been added in the Supporting Information section after the References in the revised manuscript.*

### 6. Reviewer-suggested citations

***Response:*** *We appreciate this clarification. All reviewer-suggested references have been reviewed and evaluated. Relevant publications have been cited in the revised manuscript.*

### 7. Reference list review

***Response:*** *We have carefully reviewed the entire reference list for accuracy, completeness, and retraction status. No retracted publications were identified. Minor formatting and bibliographic corrections have been made where needed, and these updates are included in the revised manuscript.*

## REVIEWER #1

### Comment 1: Background and Introduction improvements

**Comment: The background (“The understanding of recovery after critical COVID-19 beyond the first year is limited”) is too generic (L27–29). It should briefly reference existing one- or two-year follow-up gaps in research to frame the novelty more clearly.**

**The citations are comprehensive but descriptive. The authors could improve critical engagement by distinguishing methodological limitations of prior studies (e.g., smaller sample sizes, lack of frailty stratification, non-longitudinal design).**

**The narrative shifts abruptly from COVID-19 sequelae (L58–64) to ARDS comparators (L63–67). The transition sentence could better explain why comparing COVID-19 with ARDS survivors is justified.**

***Response:*** *We thank the Reviewer for this constructive comment. We have revised the Introduction to improve clarity and contextualization. Specifically, we now (1) more clearly describe the knowledge gap beyond 24 months, (2) briefly outline key methodological differences across existing long-term studies, and (3) add a transition explaining why comparisons with non-COVID ARDS are relevant.*

***Changes made:*** *Throughout Introduction (Lines 73-91).*

### Comment 2: ICU treatment variables as confounders

**Comment: Frailty and comorbidity are both considered, but their measurement overlap is not addressed. The Charlson Comorbidity Index or equivalent could have provided a more granular control for chronic disease confounding. Moreover, ICU treatment variables (ventilation duration, sedation depth, corticosteroid use) are not modeled, though they can strongly affect long-term outcomes.**

***Response:*** *The Reviewer’s comment is very wise. We acknowledge that frailty and comorbidity overlap conceptually. In our study, both the Clinical Frailty Scale (CFS) and the Charlson Comorbidity Index (CCI) were assessed and considered in the regression analyses (Tables 1 and 3–4). CFS reflects functional reserve and vulnerability to stressors, whereas CCI captures chronic disease burden. Their shared variance likely explains why comorbidity contributed less in the multivariable models when both measures were included.*

*Regarding ICU treatment variables, we agree that factors such as mechanical ventilation duration, sedation depth, and corticosteroid use may influence long-term outcomes, and we considered these in our analyses. Mechanical ventilation duration and ICU length of stay were included in the final multivariable models. However, data on sedation depth and corticosteroid dosing contained substantial missingness, which precluded their inclusion as covariates.*

## REVIEWER #2

### Comment 1: Mortality rates and generalizability

**Comment: The one-year mortality in the original cohort seems very high (48%), when compared with the 30-d mortality of 27% in Sweden, reported in a study by Chew et al. Can the authors discuss this and how a potentially higher than average mortality and probably disease severity, affect the generalizability of the results?**

*Response:
We thank the Reviewer for raising this important issue. First, the 1-year mortality in our cohort was 40% (198/498). To avoid confusion, we have revised Figure 1 to more clearly present overall mortality at each time point, including a summary box stating: “Overall mortality: 3 months: 39% (193/498) | 1 year: 40% (198/498) | 3 years: 40% (201/498).”*

*When comparing our results with the Chew et al. study, overall mortality was higher in our cohort, a difference that aligns with the markedly greater respiratory failure at ICU admission. In our cohort, 81% of patients (369/457) had severe ARDS (P/F ratio <13.3 kPa), compared with 50% in the Chew et al. cohort. To further contextualise this, we calculated 30-day mortality from ICU admission using complete mortality data. In our cohort, 163 of 496 patients (32.9%) died within 30 days. This was modestly higher than the nationwide 30-day mortality rate of 27% reported by Chew et al., consistent with the somewhat higher acute illness severity in our population.*

*Although our cohort had higher early mortality, this study focuses on long-term outcomes among survivors, which are conceptually distinct from short-term mortality estimates. The greater disease severity in our cohort may limit the generalisability of our long-term findings to settings with lower acute illness severity, and this point has been clarified in the revised Limitations section. However, several factors still support the external validity of our results: (1) complete population-based sampling across six ICUs in southern Sweden; (2) baseline demographics comparable to other critical COVID-19 cohorts (median age 66 years, 74% male, median BMI 30); and (3) excellent retention rates (87–91%), minimising attrition bias in the longitudinal analyses.*

***Changes made:*** *Results (Lines 210-212): clarification of 1-year and 3-year mortality*

*Table 1 (Line 216): addition of ARDS severity distribution showing 81% severe ARDS –*

*Limitation (Lines 378-380): expanded explanation of mortality context. Figure 1 and legend: (Lines 697-705) improved clarity of mortality flow with prominent summary box*

### Comment 2: Drop-out analysis

**Comment: Although the retention was good, almost 10% of the patients dropped out between 1- and 3-year follow-ups. Did the authors assess who these drop-outs were?**

***Response:*** *We agree with the Reviewer that it is important to describe characteristics of the dropouts. We analysed the characteristics of individuals who were lost to follow-up between the 1- and 3-year assessments, but this data may not have been presented clearly enough in the manuscript. The manuscript has now been revised. Overall, we found that the dropouts were younger, more often non-native Swedish speakers, and had slightly lower admission severity scores. Importantly, there were no substantial differences compared with retained participants regarding sex, comorbidities, frailty, ICU length of stay, or functional outcome at 1 year.*

***Changes made:*** *These findings have now been clarified in the revised manuscript, and a descriptive comparison has been added as S1 Table (Limitations section, Lines 370-372, S1 Table, Lines 770-772).*

### Comment 3: Non-native Swedish speakers

**Comment: There was a considerable number of non-native Swedish speaking patients in the cohort, how does this affect the results? Do the population norms fit well for immigrants? Are there cultural or language-related aspects in interpreting the results of the questionnaires?**

***Response:*** *This is an important and relevant question. A considerable proportion of participants were non-native Swedish speakers. By the 3-year follow-up, this proportion was smaller due to higher drop-out among these survivors, reducing the potential influence of language-related bias in the longitudinal analyses. At the same time, their inclusion increases generalisability to the real-world population, as non-native-speaking patients were common at our hospitals.*

*There are known challenges related to including non-native speakers in PROM research but excluding them would introduce greater bias. Therefore, we used validated translations of all instruments and certified interpreters when needed to minimise misinterpretation.*

*Regarding population norms, SF-36v2 was scored using US population norms, which constitute the primary reference standard for this instrument and are recommended when cohorts include participants from diverse cultural or linguistic backgrounds. This approach is widely used in long-term ICU outcome research to facilitate comparison across studies and settings. Nevertheless, cultural and linguistic factors may still influence self-reported outcomes, and population norms may not fully capture variation within immigrant subgroups.*

***Changes made****: This has now been clarified in the Limitations section (Lines 394–396).*

**Comment 4: Face-to-face vs telephone assessment**

**Comment: How would the different assessment methods, face-to-face and telephone assessment affect the results? The authors have listed this as a limitation, but since the result of impairment in the results is not unsurprising, it would be valuable to discuss the differences in more detail.**

***Response:*** *The Reviewer raises an important point. The shift from face-to-face to telephone assessments may have affected certain aspects of data collection. Telephone interviews can be more challenging for some non-native Swedish speakers, and the absence of visual cues may influence clinical judgement for interview-based measures such as GOSE. These limitations, however, are less relevant for patient-reported outcomes collected via self-administered questionnaires, as these were completed by postal mail at all three timepoints.*

***Changes made:*** *These points have now been clarified in the revised manuscript (Limitations section, Lines 374-377).*

### Comment 5: Absence of control group

**Comment: One important issue is that there are no controls in this study. Pandemic affected the whole society in most countries in many different ways, which may compromise population norms as a standard, because also individuals without critical covid or no covid at all may have experienced challenges in mental health. Please discuss.**

***Response:*** *We would like to thank the Reviewer for this very important point. We agree that the absence of a contemporary control group limits the ability to fully disentangle the long-term effects of critical COVID-19 from broader societal or pandemic-related influences. Population norms largely reflect pre-pandemic conditions, and population-level changes in mental health during the pandemic were generally small according to recent meta-analytic evidence (Sun et al., 2023). Importantly, the longitudinal design of our study allows each participant to act as their own reference, and the primary inferences therefore reflect within-person changes over time rather than absolute deviations from unaffected controls.*

***Changes made:*** *This has now been clarified in the revised manuscript (Limitations section, Lines 386-389).*

### Comment 6: Weakening connection to initial COVID-19 event

**Comment: In intensive care literature on long-term follow-up there is an inherent challenge that the connection to the initial event (here COVID-19) becomes weaker as time passes, and other life events occur and confound the results. It would be important to discuss also this aspect, that it is not clear how much these results are due to history of covid, or are they possibly a result of other challenges, such as aging, other diagnoses, etc.**

***Response:*** *The Reviewer highlights an important issue. We agree that, in long-term ICU follow-up studies, the connection between the initial critical illness and later outcomes becomes less direct over time, and intervening factors such as ageing, new or ongoing illness, and other life events may contribute to the 3-year findings. The absence of contemporary controls further limits the ability to distinguish these influences from the long-term consequences of critical COVID-19.*

*Importantly, our data support this shift. In our correlation network analysis, traditional markers of acute illness severity (SAPS 3, SOFA, comorbidity burden, mechanical ventilation duration, and ICU length of stay) showed minimal association with 3-year outcomes (GOSE, PCS, MCS). Instead, fatigue emerged as a central symptom linking physical, respiratory, and psychological domains and showing stronger associations than any acute-illness variable. This pattern suggests that post-acute mechanisms, rather than the initial severity of COVID-19, play a dominant role in long-term recovery.*

*We interpret this pattern as an important finding of the present study. The fading influence of acute illness severity and the stronger associations with persistent symptoms, such as fatigue, indicate that long-term outcomes after critical COVID-19 are shaped primarily by post-acute mechanisms rather than by the initial severity of the disease. This shift has important implications for follow-up care, highlighting the need to focus on ongoing symptom burden and functional limitations rather than on acute-phase markers****.***

***Changes made:*** *We have clarified these points in the revised Discussion (Lines 328-335).*

### Comment 7: Psychiatric diagnoses at baseline

**Comment: Did the authors record any psychiatric diagnoses at baseline? This would be important information for interpreting the results.**

***Response:*** *Systematic psychiatric diagnoses were not collected at baseline, which we acknowledge as a limitation. Pre-existing psychiatric conditions may influence vulnerability to new or worsening psychological symptoms, and such information would have strengthened the interpretation of absolute symptom levels. However, our study’s longitudinal design—with repeated assessments at 3 months, 1 year, and 3 years—allows each participant to serve as their own reference, thereby strengthening inferences about within-person changes over time. This design supports the finding that anxiety, depression, and PTSD symptoms were common and, in some cases, worsened over time. Clinically, this underscores the importance of routinely assessing mental health in the follow-up of survivors of critical COVID-19.*

***Changes made:*** *We have clarified this point in the revised manuscript (Limitations section, Lines 381-385).*

### Comment 8: Rehabilitation and psychiatric support

**Comment: Some of the patients were still receiving rehabilitation at three years. How were patients supported if they were found having psychiatric symptoms at one-year follow-up? Did they receive support, and could this have affected the results?**

***Response****: This is an important point. At the 1-year follow-up, participants who reported clinically significant symptoms of anxiety or depression were, in accordance with clinical practice, advised to seek further assessment through primary care or relevant specialist services. Additionally, a subgroup of eligible individuals participated in a related pilot randomised controlled trial evaluating CBT-ACT for post-COVID anxiety and depression (Håkansson et al., Front Psychiatry, 2022). However, participation in such interventions and subsequent treatment pathways were not captured in a structured manner, and we cannot determine how these may have influenced 3-year outcomes. The overall deterioration in psychological outcomes suggests no major influence at a group level; however, we cannot rule out that the results presented here may be slightly more favourable than would otherwise have been observed.*

***Changes made:*** *Clarified in the revised manuscript (Limitations section, Lines 381–385).*

We thank the Editor and reviewers again for their valuable feedback, which has substantially improved the manuscript.

We hope that the revised version now meets the standards for publication in PLOS ONE.

**Sincerely,**

**Ingrid Didriksson and co-authors**
