# Supplementary material for: Three-year functional, physical, and mental health outcomes after critical COVID-19: A prospective multicentre cohort study
Source: PLoS One. 2026 Feb 18;21(2):e0341319. doi: 10.1371/journal.pone.0341319 (PMC12915914; doi:10.1371/journal.pone.0341319)
Supplement: Attachment — Submitted filename: Response to Reviewers.docx [file pone.0341319.s009.docx]

**Response to Reviewers**

**Manuscript ID: PONE-D-25-38284R1**

**Title: Three-year functional, physical, and mental health outcomes after critical COVID-19: A prospective multicentre cohort study**

We thank the Academic Editor and the Reviewers for their careful evaluation of our manuscript and for their constructive and insightful comments. We have revised the manuscript accordingly and believe these revisions have strengthened clarity, methodological transparency, and the interpretation of the findings.

Below, we provide a point-by-point response to each comment raised by the reviewers. All changes made to the manuscript are highlighted in the revised version with tracked changes.

**Reviewer #1:**

**Comment 1:** Introduction remains largely descriptive rather than hypothesis-driven. While the gap in knowledge is clearly articulated, the manuscript would benefit from a clearer articulation of expected trajectories between 1 and 3 years. In addition, while the authors cite relevant two-year studies, the Introduction could more clearly differentiate critical COVID-19 survivors from general hospitalized COVID-19 populations in terms of pathophysiology and recovery expectations.

***Response:*** *We thank the Reviewer for this constructive comment. We have revised the Introduction to more explicitly articulate an a priori, hypothesis-driven framework for recovery trajectories between one and three years. Specifically, we now state that long-term outcomes beyond the first year were expected to be increasingly shaped by persistent post-COVID-19–related mechanisms and individual vulnerability, with a diminishing influence of acute ICU-related factors (Introduction, Background; Lines 90–94).*

*In addition, we have clarified the distinction between survivors of critical COVID-19 and general hospitalised COVID-19 populations by explicitly stating that recovery trajectories after critical COVID-19 should be interpreted separately from those reported in general hospitalised cohorts, and by contextualising two-year findings from mixed-severity populations using the CO-FLOW study as an illustrative example (Introduction, Background; Lines 80–82). These revisions aim to better align the cited literature with the pathophysiological context and recovery expectations specific to survivors of critical COVID-19.*

**Comment 2:** There are several methodological choices deserve deeper discussion. The transition from predominantly face-to-face follow-up at 1 year to exclusively telephone-based follow-up at 3 years introduces a non-trivial risk of measurement drift, particularly for clinician-reported outcomes such as GOSE. Although the authors acknowledge this limitation later, it would strengthen the Methods section to explicitly justify why telephone administration is considered comparable, or to cite validation studies specific to ICU or post-COVID populations.

***Response:*** *We thank the Reviewer for this important methodological comment. We have revised the Methods section to explicitly justify the use of telephone-based follow-up at three years, which was chosen as a pragmatic design decision to balance data completeness, feasibility, and participant burden, and to minimise loss to follow-up over the extended study period. While face-to-face follow-up may allow for richer clinical information, including contextual cues and involvement of next of kin, particularly relevant for clinician-reported outcomes such as the GOSE, the 3-year follow-up was deliberately designed around outcome instruments that do not require physical attendance. We further clarify that all GOSE assessments were conducted using a structured interview format and manualised scoring procedures developed to improve inter-rater reliability and ensure consistency across assessors and modes of administration. In addition, we now cite validation data demonstrating good agreement between telephone-based and face-to-face GOSE assessment (Methods; Lines 117–119, 140–145).*

*We also acknowledge in the Discussion that, although telephone-based administration of the GOSE has demonstrated good agreement with in-person assessment, face-to-face assessment may provide greater clinical depth, and formal validation specifically in post-COVID ICU populations remains limited. However, the consistency of longitudinal findings across multiple patient-reported outcome measures, which are less sensitive to mode of administration, supports the robustness of the observed trajectories despite the change in follow-up modality. Notably, all outcome measures except the GOSE were patient-reported (PROMs. These instruments demonstrated identical longitudinal trends between 1 and 3 years. This is now addressed as a study limitation (Discussion; Lines 462–466).*

**Comment 3:** The absence of a formal sample size calculation is understandable given the population-based design, but the manuscript would benefit from a brief discussion of statistical power, especially for multivariable regression analyses. Readers are left uncertain whether null findings for ICU severity variables reflect true lack of association or limited power.

***Response:*** *We thank the Reviewer for this important comment. We have now expanded the Strengths and Limitations section to explicitly address statistical power in relation to the multivariable regression analyses. We clarify that, although the overall sample size was sufficient for the primary longitudinal analyses, statistical power for multivariable regression analyses may have been limited. Accordingly, null findings, including the lack of an independent association for sex, should be interpreted with caution; however, effect estimates for ICU severity variables were generally close to null rather than highly imprecise, suggesting that the absence of association was not solely driven by limited power (Discussion; Strengths and limitations, Lines 467–471).*

**Comment 4:** The regression modeling strategy is sound but constrained. By excluding variables with higher missingness and focusing primarily on baseline characteristics, the multivariable model is structurally predisposed to identify age and frailty while under-representing social, occupational, and rehabilitation-related determinants that may be more relevant at three years.

***Response:*** *We thank the Reviewer for this thoughtful comment. We would like to clarify that several sociodemographic factors were included in the multivariable analyses but did not remain independently associated with incomplete recovery after multivariable adjustment. We have now clarified this interpretation in the Discussion, emphasising that, in this cohort, long-term outcomes at three years were more strongly shaped by individual vulnerability than by sociodemographic characteristics (Discussion; Lines 440–446).*

*Variables with substantial missingness were excluded to ensure model stability, and several clinically relevant variables could not be included simultaneously due to multicollinearity. These variables were therefore examined in univariable analyses and discussed accordingly. Rehabilitation-related factors at three years (such as fatigue, psychological symptoms, and mental HRQoL) that were assessed concurrently with functional outcome were primarily explored using correlation network analysis. This approach allowed us to characterise symptom clustering and interdependencies without implying causal direction. Rehabilitation-related aspects are addressed separately in the manuscript and discussed as part of the study’s limitations (Discussion; Strengths and limitations, Lines 478–479).*

**Comment 5:** Results would benefit from a stronger emphasis on heterogeneity. While group-level changes are clear, readers are left without a sense of which subgroups are most affected beyond age and frailty. Stratified analyses—by age group, employment status, or baseline mental health—would add important clinical nuance and help clinicians identify patients at greatest risk.

***Response:*** *We thank the Reviewer for this thoughtful and clinically relevant suggestion. Heterogeneity in long-term outcomes was explored using individual-level longitudinal analyses, multivariable regression, and correlation network modelling, which together allowed us to capture variation in recovery trajectories beyond group-level averages. To further address this point, we performed additional age-stratified analyses at the 3-year follow-up. These analyses supported our main finding that younger survivors had worse functional outcomes than older age groups, highlighting heterogeneity in recovery trajectories beyond frailty alone. The results are presented in S2 Table and integrated into the Results and Discussion (Lines 282–285, 422–423).*

*Age stratification was prioritised because age emerged as the strongest independent predictor in the multivariable model and was available without substantial missingness, whereas further stratification would have resulted in underpowered subgroups.*

*In addition, the manuscript already includes descriptive comparisons of participants with good versus incomplete recovery at 3 years, covering sociodemographic, occupational, and patient-reported characteristics. These descriptive results are presented to illustrate clinically relevant heterogeneity between outcome groups without implying statistical inference or causality.*

**Reviewer #2:** The authors have done a substantial amount of work and the manuscript has improved. They have responded to my questions and comments satisfactorily.

***Response:*** *We thank the Reviewer for their careful evaluation of the revised manuscript and for their positive feedback. We are pleased that the responses and revisions were satisfactory.*
